# Impact of upacicalcet on bone metabolism in hemodialysis patients with secondary hyperparathyroidism: a post-hoc analysis

**DOI:** 10.1093/jbmrpl/ziaf139

**Published:** 2025-08-28

**Authors:** Suguru Yamamoto, Shinji Yoneda, Hisami Yasuzawa, Junichiro James Kazama, Ichiei Narita

**Affiliations:** Division of Clinical Nephrology and Rheumatology, Niigata University Graduate School of Medical and Dental Sciences, Niigata 951-8510, Japan; Medical Affairs Department, Sanwa Kagaku Kenkyusho Co., Ltd., Nagoya 461-8631, Japan; Medical Affairs Department, Sanwa Kagaku Kenkyusho Co., Ltd., Nagoya 461-8631, Japan; Division of Nephrology and Hypertension, Fukushima Medical University, Fukushima 960-1295, Japan; Division of Clinical Nephrology and Rheumatology, Niigata University Graduate School of Medical and Dental Sciences, Niigata 951-8510, Japan

**Keywords:** Upacicalcet, secondary hyperparathyroidism, bone-specific alkaline phosphatase, PTH, tartrate-resistant acid phosphatase-5b, total type 1 procollagen-N-propeptide

## Abstract

Secondary hyperparathyroidism (SHPT) is a complication prevalent among patients undergoing hemodialysis (HD). Upacicalcet, a novel intravenous calcimimetic agent, has demonstrated efficacy in improving bone turnover by suppressing PTH production. However, the influence of baseline bone metabolism on the efficacy of calcimimetics remains unclear. Therefore, we aimed to evaluate the efficacy of upacicalcet on PTH suppression and changes in bone turnover based on bone-specific alkaline phosphatase (BAP) levels. This study involved a post-hoc analysis of data from a phase 3, placebo-controlled, double-blind trial evaluating the effect of upacicalcet in HD patients with SHPT. Patients were categorized into 3 groups based on tertiles of baseline serum BAP levels. Key biomarkers, including serum levels of intact PTH (iPTH), BAP, tartrate-resistant acid phosphatase-5b (TRACP-5b), and BAP/TRACP-5b ratio, were measured. Percentage changes from baseline in these parameters were assessed using a mixed-effects model for repeated measures. Additionally, cases of increased serum BAP levels following upacicalcet administration were investigated. A total of 103 HD patients with SHPT treated with upacicalcet were included in the analysis. Patients were categorized into low BAP (<12.8 μg/L), medium BAP (12.8-18.8 μg/L), and high BAP (>18.8 μg/L) groups. After 24 wk of upacicalcet intervention, iPTH levels decreased across all baseline BAP groups. Serum BAP and TRACP-5b levels decreased, whereas the BAP/TRACP-5b ratio increased across all groups. However, 26 (27.4%) patients exhibited increased BAP levels at week 24 relative to the levels at baseline despite the significant reduction in PTH levels. Upacicalcet treatment reduced PTH levels in HD patients with SHPT, regardless of baseline BAP levels. The concurrent increase in the BAP/TRACP-5b ratio with upacicalcet suggests that this agent may exert direct effects on bone metabolism, in addition to its role in suppressing parathyroid activity.

## Graphical Abstract

**Graphical Abstract:**
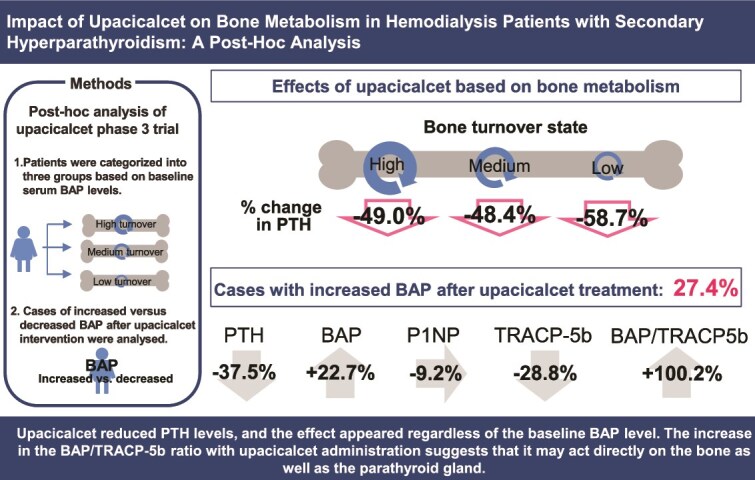

## Introduction

Secondary hyperparathyroidism (SHPT) is a significant pathological condition linked to mineral and bone disorders, often associated with CKD. Excessive production of PTH affects bone metabolism in CKD, particularly in patients with end-stage kidney disease (ESKD) undergoing hemodialysis (HD), thereby increasing the frequency of fractures.^1^

Calcimimetics suppress PTH secretion by acting on the calcium-sensing receptor of the parathyroid gland, thereby improving bone metabolism in patients with CKD.^2–6^ A systematic review and meta-analysis demonstrated that calcimimetics reduced fracture incidence in patients on dialysis.^7^ Recent basic and clinical studies suggest that calcimimetics may directly affect the parathyroid gland and the bone tissue.^8–10^

Bone metabolism in patients with CKD exhibits diverse histological patterns, ranging from high bone turnover to adynamic bone. Although bone biopsy remains the gold standard for directly assessing bone status, bone metabolism is often evaluated through clinical measurement of bone turnover markers, including bone-specific alkaline phosphatase (BAP) and tartrate-resistant acid phosphatase-5b (TRACP-5b). In SHPT, the levels of both BAP, a marker of bone formation, and TRACP-5b, a marker of bone resorption, are typically elevated. However, changes in these bone turnover markers in response to PTH may vary based on patient-specific factors, such as race and treatment regimens, suggesting differences in bone responsiveness to PTH.^11^

A phase 3, multicenter, randomized, double-blind, placebo-controlled, parallel-group study demonstrated that upacicalcet, a novel intravenous calcimimetic agent, reduced serum PTH, BAP, and TRACP-5b levels in patients with ESKD undergoing HD.^12^ These findings suggest that calcimimetics may improve bone metabolism; however, their effects on bone responsiveness may vary based on differences in baseline bone metabolism.

In the present study, a post-hoc analysis of data from the phase 3 trial was conducted, aimed at investigating changes in the levels of PTH and bone turnover markers according to baseline BAP levels in HD patients with SHPT following upacicalcet treatment. Additionally, we sought to assess the characteristics of cases, where BAP levels increased following upacicalcet treatment. We used the BAP/TRACP-5b ratio to assess bone responsiveness. We hypothesized that different baseline bone metabolism modulates the improvement in bone metabolism in response to the lowering of PTH by upacicalcet and that this agent exerts direct effects on bone metabolism.

## Materials and methods

A phase 3 study of upacicalcet was conducted across 41 sites in Japan, adhering to the principles outlined in the Declaration of Helsinki, Good Clinical Practice, and related regulations (NCT03801980). Detailed procedures of the phase 3 study have been described previously.^12^ In brief, this study was a randomized, double-blind, placebo-controlled study featuring intrapatient dose adjustments over a treatment period of 24 wk (a 21-wk dose adjustment period followed by a 3-wk evaluation period). During the study period, modifications to vitamin D receptor activators (VDRAs) and dialysate calcium concentrations were prohibited. This post-hoc analysis was approved by the Institutional Review Board of the Medical Corporation Shintokai Yokohama Minoru Clinic, Kanagawa, Japan (Approval Number: 14000021.20230209-24S141). In accordance with local regulations, an opt-out method on the website was adopted for the use of existing data.

### Patients

As previously reported,^12^ Japanese patients with CKD, aged 20 yr or older, and undergoing HD or hemodiafiltration thrice a week, with serum intact PTH (iPTH) levels >240 pg/mL for 2 consecutive weeks and serum albumin-corrected calcium (cCa) levels ≥8.4 mg/dL were eligible for inclusion in the study. No upper limits were set for serum levels of iPTH and cCa. The exclusion criteria for osteoporosis drugs prior to the screening period are as follows: use of zoledronic acid hydrate injection within the past 52 wk; use of bisphosphonates, PTH preparations, anti-receptor activator of nuclear factor-kB ligand monoclonal antibodies within the past 24 wk; and use of estrogen hormones, selective estrogen receptor modulators, or calcitonin within the past 2 wk.

### Study design

This post-hoc analysis was designed to evaluate whether variations in bone metabolism turnover could influence the effect of upacicalcet on serum levels of iPTH and bone turnover markers. Patients were divided into 3 groups based on tertiles of baseline serum BAP levels. The variables examined included iPTH, BAP, TRACP-5b, BAP/TRACP-5b ratio, phosphate (P), cCa, cCa × P product, and intact fibroblast growth factor 23 (FGF23). Correlations between iPTH and BAP, iPTH and TRACP-5b, and BAP and TRACP-5b were assessed at both baseline and week 24 in the upacicalcet treatment group. Additionally, the increase or decrease in serum BAP levels at week 24 compared to those at baseline were examined.

### Biochemical determinations

Serum levels of iPTH (electrochemiluminescence immunoassay [ECLIA]), P, and cCa were measured weekly from weeks 0 to 24. Serum alkaline phosphatase (ALP; normal range, 115-359 U/L) levels were measured every 3 wk. Serum levels of BAP (chemiluminescence enzyme immunoassay; male normal range: 3.7-20.9 μg/L, premenopausal female normal range: 2.9-14.5 μg/L, and postmenopausal female normal range: 3.8-22.6 μg/L), total type 1 procollagen-N-propeptide (total P1NP) (ECLIA; male normal range: 18.1-74.1 ng/mL, premenopausal female normal range: 16.8-70.1 ng/mL, and postmenopausal female normal range: 26.4-98.2 ng/mL) and TRACP-5b (enzyme immunoassay, male normal range: 170-590 mU/dL, and female normal range: 120-420 mU/dL) were assessed at weeks 0, 12, and 24, whereas serum intact FGF23 levels (enzyme-linked immunosorbent assay) were measured at weeks 0, 6, 12, 18, and 24. All biochemical samples were collected before the initiation of the first HD session each week. The Payne formula was used to calculate serum cCa levels for patients with serum albumin levels <4.0 g/dL.

### Statistical analysis

Baseline demographic and biochemical characteristics are presented as means with standard deviations or medians with interquartile ranges for continuous variables and as frequency counts or percentages for categorical variables. Comparison of baseline characteristics among the three groups based on tertiles of baseline serum BAP levels was conducted using a one-way analysis of variance test or Fisher’s exact test. Additionally, baseline characteristics for cases with increased or decreased BAP levels were compared using Fisher’s exact test and a two-sample *t*-test. Percentage changes in parameter values from baseline are expressed as the least squares mean (LSM) ± SE and were assessed using a mixed-effects model for repeated measures (MMRM). The model included baseline values, baseline BAP group, time (weeks), and the interaction between baseline BAP group and time as fixed effects, whereas sex, age (yr), BMI (kg/m^2^), duration of dialysis (yr), and VDRA use were incorporated as covariates. Differences in LSM estimates at week 24 among the BAP groups were evaluated using Tukey–Kramer multiple comparison adjustment or Bonferroni adjustment. Additionally, an analysis stratified by baseline serum TRACP-5b tertiles was conducted. Multivariate logistic regression analysis was performed to investigate the factors associated with the increase and decrease in serum BAP after upacicalcet intervention. Age (yr), sex, BMI (kg/m^2^), duration of dialysis (yr), and VDRA use were included as explanatory variables. Results with a *p*-value of .05 or less were considered statistically significant. All statistical analyses were performed using SAS version 9.4 (SAS Inc.).

## Results

### Efficacy of upacicalcet based on baseline BAP levels

A total of 153 patients were randomly assigned into the upacicalcet group (103 patients) and placebo group (50 patients). These patients were stratified into three tertiles based on baseline serum BAP levels: low tertile (BAP < 12.8 μg/L), medium tertile (BAP, 12.8-18.8 μg/L), and high tertile (BAP > 18.8 μg/L). The demographic and clinical characteristics of the upacicalcet and placebo groups, categorized by baseline serum BAP level tertiles, are summarized in Table 1 and Table S1, respectively. In the upacicalcet group, significant differences in baseline characteristics were observed among the baseline BAP tertiles in terms of sex, age, dry weight, VDRA use, and serum levels of P, cCa × P product, ALP, BAP, total P1NP, TRACP-5b, BAP/TRACP-5b ratio, and FGF23. However, no significant differences were observed in serum iPTH levels (Table 1).

**Table 1:** Baseline characteristics of the upacicalcet group categorized by baseline serum BAP level tertiles.

**Parameter**	**Baseline serum BAP**			***p*-value**
	**Low** **(<12.8 μg/L)**	**Medium** **(12.8-18.8 μg/L)**	**High** **(>18.8 μg/L)**	
	**(*n* = 32)**	**(*n* = 37)**	**(*n* = 34)**	
**Sex (male), *n* (%)**	28 (87.5)	30 (81.1)	19 (55.9)	.009
**Age, years**	55.9±12.2	66.2±10.7	64.4±12.9	.001
**Dry weight, kg**	67.27±12.70	60.32±9.82	58.89±16.98	.029
**Body mass index, kg/m^2^**	24.08±3.69	22.80±3.02	22.60±4.13	.205
**Primary disease, *n* (%)**				
** Chronic glomerulonephritis**	11 (34.4)	17 (45.9)	8 (23.5)	.407
** Diabetes kidney disease**	10 (31.3)	10 (27.0)	13 (38.2)	
** Nephrosclerosis**	4 (12.5)	5 (13.5)	4 (11.8)	
** Polycystic kidney**	2 (6.3)	0 (0.0)	0 (0.0)	
** Other, including unknown**	5 (15.6)	5 (13.5)	9 (26.5)	
**Duration of dialysis, years**	7.49±6.83	11.10±9.27	11.70±8.63	.092
**Dialysate calcium concentration, *n* (%)**				
** 2.5 mEq/L**	9 (28.1)	14 (37.8)	9 (26.5)	.500
** 2.75 mEq/L**	15 (46.9)	10 (27.0)	14 (41.2)	
** 3.0 mEq/L**	8 (25.0)	13 (35.1)	11 (32.4)	
**Phosphate binder use, *n* (%)**	29 (90.6)	35 (94.6)	31 (91.2)	.812
**Vitamin D receptor activator use, *n* (%)**	29 (90.6)	23 (62.2)	24 (70.6)	.021
**Prior calcimimetic use, *n* (%)**	18 (56.3)	21 (56.8)	19 (55.9)	1.000
**Serum intact PTH, pg/mL**	359.0 (299.0, 445.5)	392.0 (310.0, 466.0)	366.5 (300.0, 491.0)	.423
**Serum corrected calcium, mg/dL**	9.37±0.62	9.30±0.73	9.36±0.69	.890
**Serum phosphate, mg/dL**	6.44±1.62	6.05±1.23	5.39±1.01	.005
**Serum corrected calcium × phosphate, mg^2^/dL^2^**	60.273±15.061	56.204±12.029	50.691±11.119	.011
**Serum ALP, U/L**	185.5 (161.5, 216.0)	228.0 (196.0, 301.0)	345.5 (288.0, 490.0)	<.001
**Serum BAP, μg/L**	10.70 (9.25, 12.10)	15.50 (13.90, 16.90)	25.65 (20.90, 32.10)	<.001
**Serum total P1NP, ng/mL**	217.5 (157.0, 314.0)	360.0 (289.0, 449.0)	511.0 (370.0, 653.0)	<.001
**Serum TRACP-5b, mU/dL**	394.5 (271.5, 567.0)	723.0 (552.0, 902.0)	914.0 (745.0, 1130.0)	<.001
**Serum BAP/TRACP-5b ratio**	0.0257 (0.0188, 0.0336)	0.0231 (0.0178, 0.0259)	0.0317 (0.0205, 0.0397)	.002
**Serum fibroblast growth factor 23, pg/mL**	15 600 (5880, 33 350)	7390 (2590, 22 500)	3850 (662, 11 600)	.007

Data are shown as number (%), mean ± SD or median (25th percentile, 75th percentile).

Fisher’s exact test or one-way ANOVA was used for comparisons between groups.

Abbreviations: ALP, alkaline phosphatase; BAP, bone-specific alkaline phosphatase; P1NP, type 1 procollagen-N-propeptide; TRACP-5b, tartrate-resistant acid phosphatase-5b.

**Figure 1:**
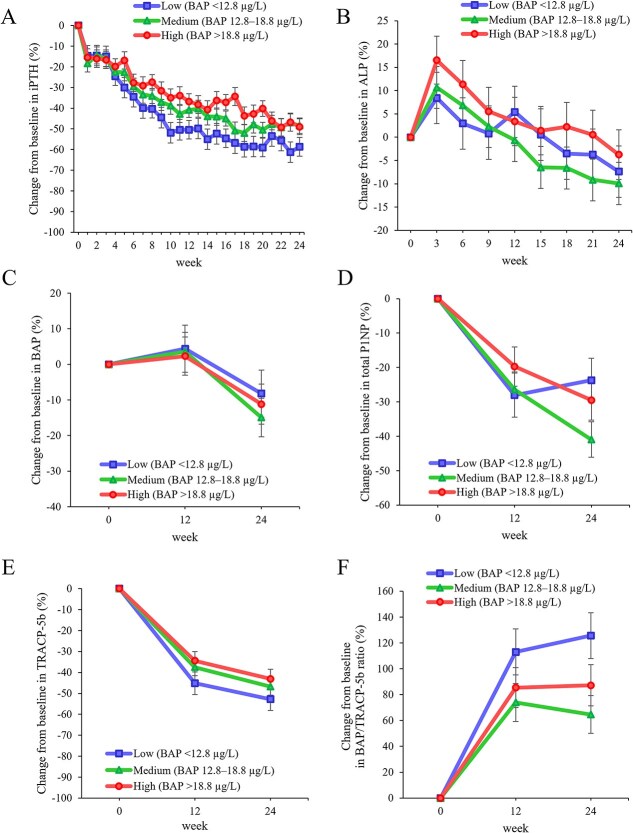
Percentage changes from baseline in serum levels of iPTH (A), ALP (B), BAP (C), total P1NP (D), TRACP-5b (E), and BAP/TRACP-5b ratio (F) by baseline serum BAP level tertiles in the upacicalcet group. Data are shown as least square mean ± SE. Abbreviations: ALP, alkaline phosphatase; BAP, bone-specific alkaline phosphatase; iPTH, intact PTH; P1NP, type 1 procollagen-N-propeptide; TRACP-5b, tartrate-resistant acid phosphatase-5b.

Following 24 wk of upacicalcet intervention, serum iPTH levels decreased across all tertiles: −58.7 ± 4.5%, −48.4 ± 3.7%, and −49.0 ± 3.8% in the low, medium, and high tertiles, respectively (Figure 1A). A trend toward a greater decrease in serum iPTH levels was observed in the low tertile compared with that in the medium (10.3 ± 5.4%, *p* = .182) and high tertiles (9.7 ± 5.6%, *p* = .260) (Table S2). In the medium and high tertiles, serum ALP levels increased at week 3 and subsequently decreased. In contrast, serum ALP levels in the low tertile did not increase. In all tertiles, serum levels of BAP, total P1NP and TRACP-5b decreased, and the serum BAP/TRACP-5b ratio increased (Figure 1B-F). These changes were not significantly different among the three tertiles at week 24, except for BAP/TRACP-5b ratio (Table S2). Additionally, no significant differences were observed in the serum levels of P, cCa, cCa × P product, or FGF23 among the three tertiles (Figure S1 and Table S2). In the placebo group, no differences were observed in the serum levels of iPTH, ALP, BAP, total P1NP, TRACP-5b, BAP/TRACP-5b ratio, cCa, P × cCa product, or FGF23 across the three tertiles. However, serum P levels showed a trend toward a difference between the high and medium tertiles (−17.1 ± 7.3%, *p* = .051) (Figure S2). Additionally, percentage changes in serum iPTH, ALP, BAP, total P1NP, TRACP-5b, and BAP/TRACP-5b ratio stratified by baseline TRACP-5b tertiles are shown in Table S3 and Figure S3, and these changes showed similar trends to those observed with BAP tertile stratification.

### Correlations between iPTH and BAP, iPTH and TRACP-5b, and BAP and TRACP-5b

**Figure 2:**
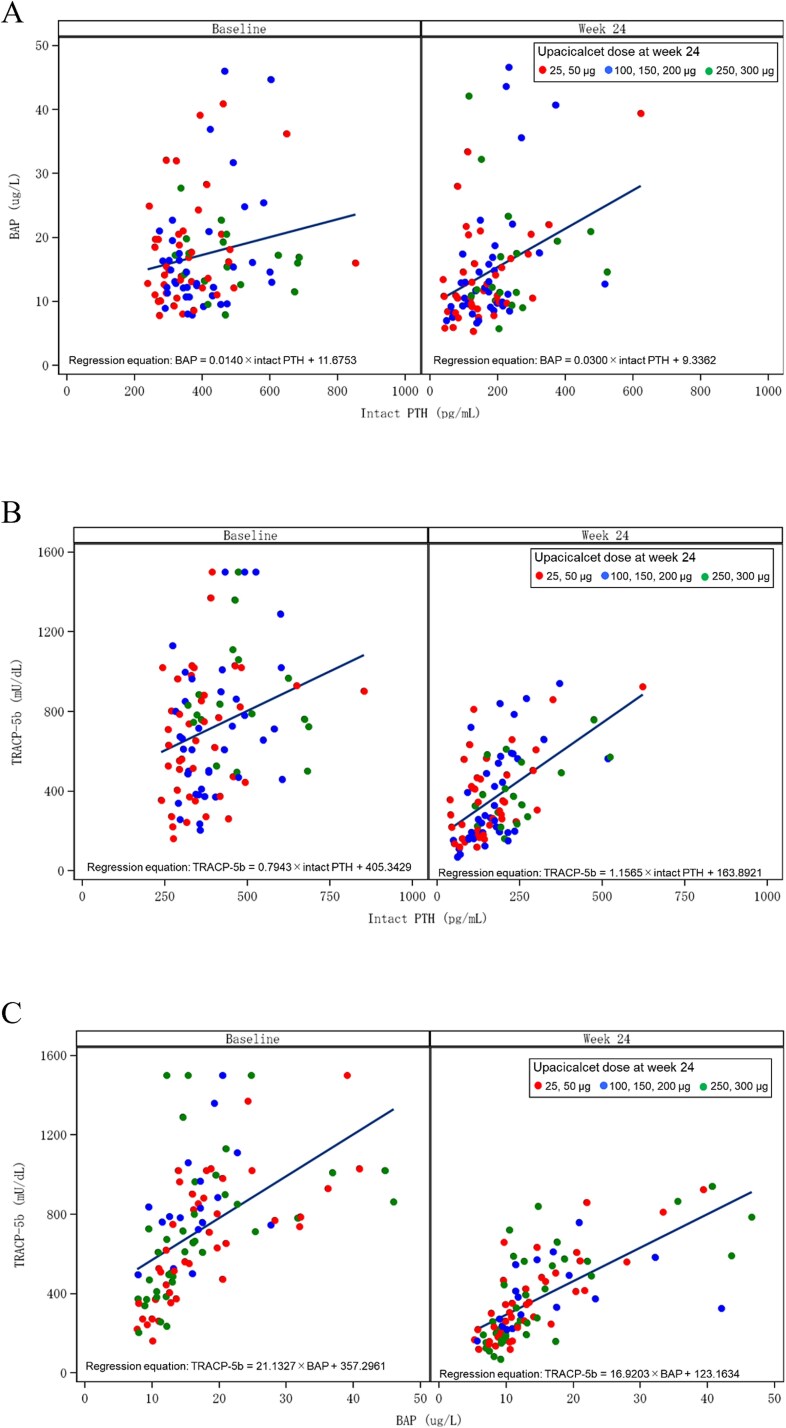
The correlations between iPTH and BAP (A), iPTH and TRACP-5b (B), and BAP and TRACP-5b (C) before and after upacicalcet intervention. Abbreviations: BAP, bone-specific alkaline phosphatase; iPTH, intact PTH; TRACP-5b, tartrate-resistant acid phosphatase-5b.

The correlations among iPTH, BAP, and TRACP-5b before and after upacicalcet intervention are depicted in Figure 2. Following upacicalcet intervention, serum iPTH levels exhibited a leftward shift, whereas serum BAP and TRACP-5b levels demonstrated a downward shift (Figure 2A and B). In the relationship between serum BAP and TRACP-5b, serum BAP levels exhibited a slight leftward shift, whereas serum TRACP-5b levels exhibited a substantial downward shift following upacicalcet intervention (Figure 2C).

### Cases with increased BAP levels after upacicalcet intervention

**Figure 3:**
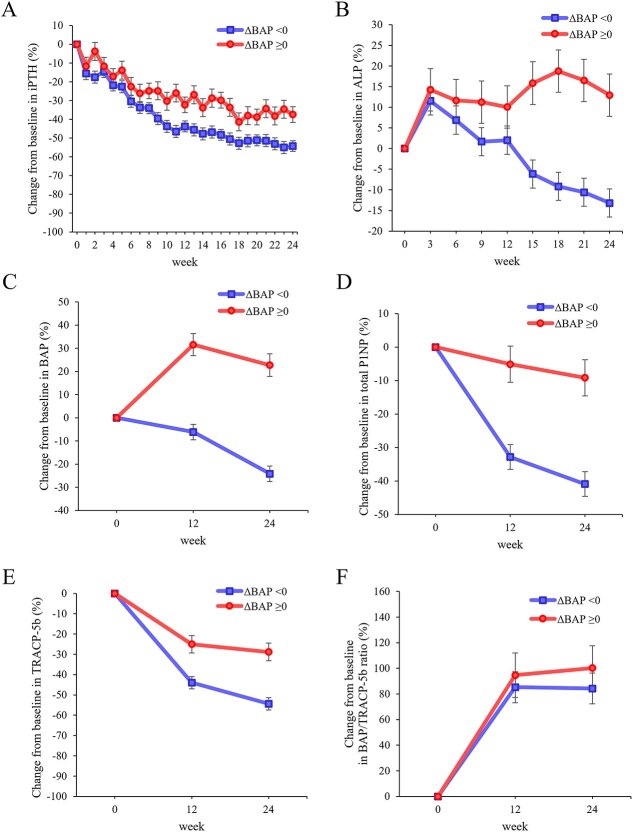
Percentage changes from baseline in serum levels of iPTH (A), ALP (B), BAP (C), total P1NP (D), TRACP-5b (E), and BAP/TRACP-5b ratio (F) in patients with increased or decreased serum BAP levels at week 24 in the upacicalcet group. Data are shown as least square mean ± SE. Abbreviations: ALP, alkaline phosphatase; BAP, bone-specific alkaline phosphatase; iPTH, intact PTH; P1NP, type 1 procollagen-N-propeptide; TRACP-5b, tartrate-resistant acid phosphatase-5b.

Ninety-five patients completed upacicalcet intervention for 24 wk. Among these patients, 26 (27.4%) exhibited increased serum BAP levels at week 24 compared with the levels at baseline. Notably, patients with increased serum BAP levels were younger than those with decreased BAP levels (Table 2). At week 24, the doses of upacicalcet administered to patients with increased and decreased serum BAP levels were 148.1 ± 103.9 μg/session and 110.4 ± 87.4 μg/session, respectively (*p* = .079). In patients with increased BAP levels, serum iPTH and TRACP-5b levels decreased significantly to 37.5 ± 4.2% (*p* < .001) and 28.8 ± 4.3% (*p* < .001), respectively, whereas serum ALP level was significantly increased to 12.9 ± 5.1% (*p* = .012) and serum total P1NP level was not significantly changed (*p* = .093) (Figure 3A-E). Furthermore, patients with increased serum BAP levels exhibited significantly lower percentage changes in serum levels of iPTH (difference; 16.8 ± 4.8%, *p* < .001), ALP (difference; 26.1 ± 5.9%, *p* < .001), total P1NP (difference; 31.8 ± 6.2%, *p* < .001), and TRACP-5b (difference; 25.5 ± 5.0%, *p* < .001) at week 24 than patients with decreased serum BAP levels. However, no significant difference in the change in serum BAP/TRACP-5b ratio was observed between the two groups (difference; 15.9 ± 19.9%, *p* = .426) (Figure 3F). Multivariate logistic regression analysis revealed that age was significantly associated with increased serum BAP levels (odds ratio 0.944 [0.906-0.983]; *p* = .006; Table 3).

**Table 2:** Baseline characteristics of patients with increased or decreased serum BAP levels at week 24 in the upacicalcet group.

Parameter	ΔBAP at week 24		*p-*value
	ΔBAP ≥ 0	ΔBAP < 0	
	(*n* = 26)	(*n* = 69)	
**Sex (male), *n* (%)**	20 (76.9%)	52 (75.4%)	1.000
**Age, years**	55.7±14.3	64.1±11.6	.004
**Dry weight, kg**	61.28±14.02	63.24±13.81	.540
**BMI, kg/m^2^**	22.75±4.15	23.41±3.57	.445
**Primary disease, *n* (%)**			
** Chronic glomerulonephritis**	6 (23.1%)	26 (37.7%)	.369
** Diabetes kidney disease**	8 (30.8%)	23 (33.3%)	
** Nephrosclerosis**	5 (19.2%)	7 (10.1%)	
** Polycystic kidney**	0 (0.0%)	2 (2.9%)	
** Other, including unknown**	7 (26.9%)	11 (15.9%)	
**Duration of dialysis, years, *n* (%)**	11.95±9.07	9.48±8.26	.210
**Dialysate calcium concentration, *n* (%)**			
** 2.5 mEq/L**	10(38.5%)	22(31.9%)	.711
** 2.75 mEq/L**	10(38.5%)	25(36.2%)	
** 3.0 mEq/L**	6(23.1%)	22(31.9%)	
**Phosphate binder use, *n* (%)**	23(88.5%)	65(94.2%)	.387
**Vitamin D receptor activator use, *n* (%)**	16(61.5%)	57(82.6%)	.054
**Prior calcimimetic use, *n* (%)**	16(61.5%)	40(58.0%)	.818
**Serum intact PTH, pg/mL**	392.5 (310.0, 474.0)	364.0 (310.0, 445.0)	.487
**Serum corrected calcium, mg/dL**	9.42±0.75	9.32±0.62	.501
**Serum phosphate, mg/dL**	5.95±1.26	6.03±1.39	.817
**Serum corrected calcium × phosphate, mg^2^/dL^2^**	56.422±13.742	56.079±12.884	.910
**Serum ALP, U/L**	266.5 (227.0, 338.0)	220.0 (188.0, 292.0)	.357
**Serum BAP, μg/L**	16.30 (11.00, 20.50)	14.60 (12.20, 19.70)	.823
**Serum total P1NP, ng/mL**	346.5 (211.0, 510.0)	331.0 (243.0, 449.0)	.464
**Serum TRACP-5b, mU/dL**	770.5 (495.0, 929.0)	673.0 (472.0, 902.0)	.990
**Serum BAP/TRACP-5b ratio**	0.0238 (0.0186, 0.0372)	0.0249 (0.0183, 0.0311)	.719
**Serum fibroblast growth factor 23, pg/mL**	9805 (3770, 28 900)	7860 (2200, 17 600)	.508

Data are shown as number (%), mean ± SD or median (25th percentile, 75th percentile).

Fisher’s exact test or two-sample t-test was used for comparisons between groups.

Abbreviations: ALP, alkaline phosphatase; BAP, bone-specific alkaline phosphatase; P1NP, type 1 procollagen-N-propeptide; TRACP-5b, tartrate-resistant acid phosphatase-5b.

**Table 3:** Multivariate logistic regression analysis for the increase in serum BAP levels.

	Odds ratio	95% confidence interval	*p*-value
**Age, years**	0.944	[0.906, 0.983]	.006
**Sex (male)**	1.306	[0.387, 4.405]	.667
**Duration of dialysis, years**	1.035	[0.975, 1.098]	.256
**Body mass index, kg/m^2^**	0.951	[0.834, 1.084]	.449
**Vitamin D receptor activator, no use**	2.664	[0.898, 7.896]	.077

## Discussion

In this study, we hypothesized that baseline bone metabolism could modulate the efficacy of upacicalcet and could exert direct effects on bone metabolism. Upacicalcet significantly reduced serum PTH levels in HD patients with SHPT, regardless of the baseline serum BAP levels. Additionally, the treatment decreased serum levels of BAP, total P1NP, and TRACP-5b and increased the serum BAP/TRACP-5b ratio. Moreover, serum ALP levels transiently increased in the medium and high tertiles. Notably, a subset of patients exhibited increased BAP levels following upacicalcet administration.

In patients with CKD, bone responsiveness to PTH is impaired owing to the effects of uremic toxins, contributing to the pathophysiology of SHPT.^11^^,^^13^^,^^14^ In a study examining bone metabolism markers and bone biopsy findings in patients with CKD, histological bone formation rates were notably low when PTH levels were within the normal range, necessitating 1.9 times the upper limit of normal PTH levels to achieve a normal bone formation rate.^15^ However, bone responsiveness to PTH may vary across different cases. A comparative analysis of bone metabolism markers between Europe and Japan indicated that BAP and TRACP-5b levels in Japanese patients undergoing dialysis were lower than those in Belgian patients for any given PTH level.^16^ This result suggests that bone responsiveness to PTH in Japanese patients on dialysis is lower than that in Belgian patients on dialysis. Furthermore, an international comparison of the PTH/ALP ratio from the Dialysis Outcomes and Practice Patterns Study demonstrated a higher ratio in Japanese patients on dialysis than in patients from Europe and the U.S.^1^ Moreover, a cross-sectional study revealed that the association between PTH and TRACP-5b differed between patients on HD receiving calcimimetics and those who were not.^10^ Although these findings remain controversial, bone responsiveness to PTH may vary based on race and region, PTH levels, and SHPT medications. Such differences may ultimately influence the effect of calcimimetics on bone metabolism in HD patients with SHPT.

In this study, the effects of upacicalcet on changes in bone turnover markers based on differences in baseline BAP levels were investigated. In the group with high BAP levels, which reflects a high bone turnover state, upacicalcet decreased PTH levels, similar to that in the other groups. This result suggests that upacicalcet can effectively control SHPT, regardless of baseline bone metabolism status. In contrast, in the group with low baseline BAP levels, a trend toward a greater reduction in PTH levels with upacicalcet compared to those in the other groups was observed. The low BAP level group had a higher proportion of patients administered VDRA than the other groups. Notably, active vitamin D has been suggested to increase calcium-sensing receptor expression in the parathyroid gland,^17^ potentially enhancing the efficacy of upacicalcet.

Upacicalcet transiently increased ALP in the medium and high tertiles but not significantly in the low tertile. Transient increases in ALP and BAP have been observed after calcimimetic treatment and parathyroidectomy, which may be due to hungry bone or improved bone metabolism due to lowering PTH.^18^^,^^19^ In the low tertile, serum PTH levels were decreased to the same extent as in the other tertiles, but serum ALP levels were not transiently elevated, which may be influenced by the baseline bone metabolism status. Upacicalcet reduced the serum levels of both BAP and TRACP-5b across all BAP groups. This effect was more pronounced for TRACP-5b, resulting in a significant increase in the BAP/TRACP-5b ratio. This result suggests that upacicalcet suppressed abnormal bone turnover, shifting it toward bone formation. Supporting this, analysis stratified by baseline TRACP-5b tertiles showed similar trends in bone turnover markers. The underlying mechanism likely involves both its calcimimetic action on the parathyroid gland, reducing PTH secretion, and a direct effect on the bone. Calcimimetics have been demonstrated to increase bone formation rates in animal models of kidney damage, even after parathyroidectomy.^8^ Furthermore, they have been reported to induce Erk1/2 phosphorylation in osteoblasts, thereby promoting the expression of osteogenic genes and enhancing matrix mineralization. In vitro studies have also demonstrated that cinacalcet inhibits tartrate-resistant acid phosphatase activity and hydroxyapatite resorption in human osteoclasts.^9^ Clinically, cinacalcet has been reported to improve bone histology in patients with SHPT by reducing PTH levels and bone turnover markers.^20^^,^^21^ Etelcalcetide has also been shown to improve bone density and trabecular bone scores.^22^ Whereas cinacalcet has been reported to increase bone turnover markers despite PTH suppression, suggesting a potential direct effect on bone metabolism independent of PTH.^23^ It is unclear whether the promotion of bone formation is specific to upacicalcet; further studies are needed to clarify potential differences among calcimimetics. Moreover, a systematic review and meta-analysis revealed that calcimimetics reduced fracture incidence in dialysis patients with SHPT.^7^ Therefore, calcimimetics may improve bone metabolism by acting on both the parathyroid gland and bone, thereby lowering fracture incidence in patients on dialysis.

Notably, a subset of patients exhibited increased BAP levels following upacicalcet administration, with younger age being a distinguishing characteristic of this population. The response of bone to calcimimetics may differ between younger and older patients. A previous study demonstrated that the effect of cinacalcet on cardiovascular disease was weak in participants aged <65 yr.^2^ Another possibility is that the effect of upacicalcet on bone metabolism was insufficient in this population, even with a 37.5% reduction in PTH levels, as the placebo group also exhibited increased BAP levels over the 24-wk observation period. The mean upacicalcet dose for patients with increased serum BAP levels at week 24 was 148.1 ± 103.9 μg/session, suggesting that a higher calcimimetic dose might be necessary to effectively regulate bone metabolism in this subgroup. Additionally, the increase in serum ALP levels was maintained in patients with increased BAP levels, whereas the serum ALP levels decreased after a transient increase in patients with decreased BAP levels. This suggests that the effects of hungry bones may be more likely to be maintained in patients with increased BAP levels. Although ALP reflects not only bone metabolism but can also be influenced by liver dysfunction, no investigational drug-related adverse events classified as hepatobiliary disorders were observed in this study. Moreover, upacicalcet may exert a more direct effect on the bone than on the parathyroid gland in these patients. In this study, symptomatic hypocalcemia was not observed. Only two patients had transient cCa levels <7.5 mg/dL—the threshold for temporary discontinuation of calcimimetics according to Japanese drug labels—but both resumed treatment after temporary interruption and maintained levels ≥7.5 mg/dL thereafter.^12^ Furthermore, in patients with increased BAP levels, the levels of serum total P1NP, produced by osteoblasts during the proliferative phase, did not change significantly. These findings support the interpretation that bone mineralization may be enhanced in these patients. Observational studies have shown that elevated ALP levels are associated with increased fracture risk.^1^ However, interpreting BAP dynamics during calcimimetic therapy requires caution, and utilizing the BAP/TRACP-5b ratio may offer a more accurate reflection of bone metabolism. An increase in BAP levels and the BAP/TRACP-5b ratio with SHPT treatment could indicate improvements in bone metabolism.

This study has several limitations that warrant further consideration. First, the population was limited to only Japanese patients undergoing HD. Notably, serum PTH is typically maintained at lower levels in Japan than in other countries. In fact, the target range for serum iPTH levels recommended by the Japanese Society for Dialysis Therapy (JSDT) guidelines is 60-240 pg/mL.^24^ In addition, ethnic differences in bone responsiveness to PTH have been reported. Therefore, these region- and population-specific factors may limit the generalizability of our findings to other ethnic groups and dialysis populations. Therefore, further studies involving other ethnic groups and populations undergoing HD are required to generalize the efficacy of upacicalcet. Second, the intervention period was only 24 wk. Third, as bone biopsy and BMD were not assessed, it remains uncertain how the biochemical improvements observed in this study reflect actual changes in bone quality or the risk of fracture. Fourth, although BAP was used as a surrogate marker of bone turnover, there are no well-established or validated cut-off values for BAP specific to the Japanese population.^25^ Fifth, as this was an exploratory post-hoc analysis, it is subject to inherent limitations, including the potential for selection bias and the lack of adjustment for confounding factors. Therefore, the findings should be interpreted with caution, and further prospective studies are needed to confirm these observations. This study was designed to verify the superiority of upacicalcet over placebo, with the primary outcome emphasizing reductions in serum PTH levels. Therefore, longer-term studies are needed to examine the effect of upacicalcet on the incidence of fracture in HD patients with SHPT.

Nevertheless, the strength of this study lies in its design as a sub-analysis of a randomized, placebo-controlled trial, which clearly demonstrated the effects of upacicalcet and enabled detailed monitoring of the time-dependent changes in the expression of bone metabolism markers. This design allowed for verifying the direct effects of upacicalcet on bone metabolism.

In conclusion, upacicalcet significantly reduced PTH levels in HD patients with SHPT, regardless of baseline BAP levels, including those with high BAP levels. Additionally, the significant increase in the BAP/TRACP-5b ratio suggests notable improvement in bone metabolism. The observation that some patients exhibited an increase in BAP levels after upacicalcet administration suggests that this agent may act directly on the bone, in addition to its action on the parathyroid gland. Namely, the effective suppression of PTH by upacicalcet induces a favorable response in bone, characterized by reduced bone resorption and concomitant improvement in bone mineralization. However, long-term observations are necessary to determine whether this effect is associated with fracture prevention.

## Supplementary Material

Supplementary_material_20250811_ziaf139_Clean_20250902

## Data Availability

The dataset from this study is not available in any open data repository. The purpose of use of the dataset is limited to application for marketing authorization and article publication, and any other use of the dataset may exceed the limitations of the participant’s informed consent. Requests for disclosure of datasets from this study should be addressed to Sanwa Kagaku Kenkyusho, which is the funder and data use rights holder of this study, informing them of the purpose of data use.
