# Supplementary material for: Impact of upacicalcet on bone metabolism in hemodialysis patients with secondary hyperparathyroidism: a post-hoc analysis
Source: JBMR Plus. 2025 Aug 28;9(10):ziaf139. doi: 10.1093/jbmrpl/ziaf139 (PMC12448387; doi:10.1093/jbmrpl/ziaf139)
Supplement: Supplementary_material_20250811_ziaf139_Clean_20250902 [file supplementary_material_20250811_ziaf139_clean_20250902.docx]

**Supplementary Table S1**

Baseline characteristics of the placebo group categorized by baseline serum BAP level tertiles.

| Parameter |  | Baseline serum BAP | | | | | | | | | | |  | *p*-value |
| --- | --- | --- | --- | --- | --- | --- | --- | --- | --- | --- | --- | --- | --- | --- |
|  |  | Low  (<12.8 µg/L) | | |  | Medium  (12.8–18.8 µg/L) | | |  | High  (>18.8 µg/L) | | |  |  |
|  |  | (n=18) | | |  | (n=17) | | |  | (n=15) | | |  |  |
| Sex (male), n (%) |  | 18 (100.0) | | |  | 13 (76.5) | | |  | 8 (53.3) | | |  | 0.002 |
| Age, years |  | 60.1 | ± | 15.0 |  | 68.9 | ± | 13.1 |  | 69.1 | ± | 10.9 |  | 0.082 |
| Dry weight, kg |  | 67.23 | ± | 12.71 |  | 57.36 | ± | 9.53 |  | 53.31 | ± | 10.00 |  | 0.002 |
| Body mass index, kg/m^2^ |  | 24.54 | ± | 4.08 |  | 22.44 | ± | 3.64 |  | 21.43 | ± | 2.94 |  | 0.049 |
| Primary disease, n (%) |  |  |  |  |  |  |  |  |  |  |  |  |  |  |
| Chronic glomerulonephritis |  | 7 (38.9) | | |  | 8 (47.1) | | |  | 9 (60.0) | | |  | 0.383 |
| Diabetes kidney disease |  | 4 (22.2) | | |  | 5 (29.4) | | |  | 1 (6.7) | | |  |  |
| Nephrosclerosis |  | 2 (11.1) | | |  | 0 (0.0) | | |  | 3 (20.0) | | |  |  |
| Polycystic kidney |  | 1 (5.6) | | |  | 0 (0.0) | | |  | 0 (0.0) | | |  |  |
| Other, including unknown |  | 4 (22.2) | | |  | 4 (23.5) | | |  | 2 (13.3) | | |  |  |
| Duration of dialysis, years |  | 7.29 | ± | 6.98 |  | 10.95 | ± | 7.37 |  | 5.50 | ± | 4.05 |  | 0.057 |
| Dialysate calcium concentration, n (%) |  |  |  |  |  |  |  |  |  |  |  |  |  |  |
| 2.5 mEq/L |  | 4 (22.2) | | |  | 5 (29.4) | | |  | 5 (33.3) | | |  | 0.921 |
| 2.75 mEq/L |  | 9 (50.0) | | |  | 7 (41.2) | | |  | 5 (33.3) | | |  |  |
| 3.0 mEq/L |  | 5 (27.8) | | |  | 5 (29.4) | | |  | 5 (33.3) | | |  |  |
| Phosphate binder use, n (%) |  | 17 (94.4) | | |  | 16 (94.1) | | |  | 13 (86.7) | | |  | 0.671 |
| Vitamin D receptor activator use, n (%) |  | 16 (88.9) | | |  | 15 (88.2) | | |  | 12 (80.0) | | |  | 0.774 |
| Prior calcimimetics use, n (%) |  | 12 (66.7) | | |  | 8 (47.1) | | |  | 7 (46.7) | | |  | 0.406 |
| Serum intact parathyroid hormone, pg/mL |  | 349.0  (306.0, 449.0) | | |  | 366.0  (281.0, 411.0) | | |  | 397.0  (323.0, 561.0) | | |  | 0.118 |
| Serum corrected calcium, mg/dL |  | 9.32 | ± | 0.80 |  | 9.53 | ± | 0.65 |  | 9.35 | ± | 0.79 |  | 0.680 |
| Serum phosphate, mg/dL |  | 7.02 | ± | 1.61 |  | 5.71 | ± | 1.41 |  | 5.61 | ± | 1.24 |  | 0.009 |
| Serum corrected calcium x phosphate, mg^2^/dL^2^ |  | 64.977 | ± | 14.037 |  | 54.674 | ± | 15.484 |  | 52.901 | ± | 14.593 |  | 0.043 |
| Serum ALP, U/L |  | 194.5  (154.0, 258.0) | | |  | 238.0  (219.0, 291.0) | | |  | 355.0  (281.0, 417.0) | | |  | <0.001 |
| Serum BAP, µg/L |  | 10.85  (8.00, 11.90) | | |  | 16.10  (15.20, 18.10) | | |  | 26.60  (23.10, 33.80) | | |  | <0.001 |
| Serum total P1NP, ng/mL |  | 217.0  (165.0, 287.0) | | |  | 378.0  (283.0, 390.0) | | |  | 472.0  (334.0, 1100.0) | | |  | <0.001 |
| Serum TRACP-5b, mU/dL |  | 419.5  (319.0, 731.0) | | |  | 717.0  (574.0,841.0) | | |  | 826.0  (717.0, 1410.0) | | |  | 0.002 |
| Serum BAP/ serum TRACP-5b ratio |  | 0.0213  (0.0165, 0.0317) | | |  | 0.0248  (0.0152, 0.0300) | | |  | 0.0329  (0.0242, 0.0411) | | |  | 0.173 |
| Serum fibroblast growth factor 23, pg/mL |  | 17750  (8440, 38700) | | |  | 6110  (4320, 11100) | | |  | 3700  (954, 33900) | | |  | 0.102 |

Data are shown as number (%), mean ± standard deviation or median (25th percentile, 75th percentile).

Fisher's exact test or one-way ANOVA test was used for comparisons between groups.

ALP, alkaline phosphatase; BAP, bone-specific alkaline phosphatase; P1NP, type 1 procollagen-N-propeptide; TRACP-5b, tartrate-resistant acid phosphatase-5b.

**Supplementary Table S2**

Differences in least squares mean estimates at week 24 among the BAP groups in the upacicalcet group

|  | iPTH | | | ALP | | | BAP | | | total P1NP | | | TRACP-5b | | | BAP/TRACP-5b ratio | | | P | | | cCa | | | P x cCa | | | FGF23 | | |  |
| --- | --- | --- | --- | --- | --- | --- | --- | --- | --- | --- | --- | --- | --- | --- | --- | --- | --- | --- | --- | --- | --- | --- | --- | --- | --- | --- | --- | --- | --- | --- | --- |
| Medium BAP tertile  *vs.*  Low BAP tertile | 10.3 | ± | 5.4 | -2.5 | ± | 6.7 | -6.7 | ± | 7.9 | -17.2 | ± | 7.7 | 6.0 | ± | 6.3 | -61.0 | ± | 21.4^a^ | -6.5 | ± | 5.3 | -0.3 | ± | 1.4 | -6.3 | ± | 5.0 | -7.1 | ± | 19.9 | |
| High BAP tertile  *vs.*  Low BAP tertile | 9.7 | ± | 5.6 | 3.6 | ± | 7.8 | -3.0 | ± | 8.2 | -5.8 | ± | 8.7 | 9.7 | ± | 7.2 | -38.5 | ± | 22.4 | -9.6 | ± | 5.6 | -0.1 | ± | 1.5 | -8.3 | ± | 5.3 | -0.8 | ± | 21.2 | |
| High BAP tertile  *vs.*  Medium BAP tertile | -0.6 | ± | 5.1 | 6.2 | ± | 7.0 | 3.8 | ± | 7.5 | 11.5 | ± | 7.5 | 3.7 | ± | 5.9 | 22.6 | ± | 21.3 | -3.1 | ± | 5.2 | 0.2 | ± | 1.4 | -1.9 | ± | 4.9 | 6.3 | ± | 19.5 | |

Data are shown as least squares mean ± standard error.

Tukey-Kramer multiple comparison adjustment or Bonferroni adjustment was used for comparisons between groups. ^a^ P<0.05

Low BAP tertile, BAP <12.8 µg/L; medium BAP tertile, BAP 12.8–18.8 µg/L; and high BAP tertile, BAP >18.8 µg/L

BAP, bone-specific alkaline phosphatase; ALP, alkaline phosphatase; P1NP, type 1 procollagen-N-propeptide; iPTH, intact parathyroid hormone; TRACP-5b, tartrate-resistant acid phosphatase-5b; P, phosphate; cCa, corrected calcium; FGF23, fibroblast growth factor 23

**Supplementary Table S3**

Baseline characteristics of the upacicalcet group categorized by baseline serum TRACP-5b level tertiles.

| Parameter |  | Baseline serum TRACP-5b | | | | | | | | | | |  | *p*-value |
| --- | --- | --- | --- | --- | --- | --- | --- | --- | --- | --- | --- | --- | --- | --- |
|  |  | Low  (<561 mU/dL) | | |  | Medium  (561–826 mU/dL) | | |  | High  (>826 mU/dL) | | |  |  |
|  |  | (n=35) | | |  | (n=32) | | |  | (n=36) | | |  |  |
| Sex (male), n (%) |  | 30 (85.7) | | |  | 25 (78.1) | | |  | 22 (61.1) | | |  | 0.060 |
| Age, years |  | 57.4 | ± | 12.7 |  | 66.0 | ± | 11.8 |  | 64.0 | ± | 11.9 |  | 0.012 |
| Dry weight, kg |  | 69.86 | ± | 16.30 |  | 58.51 | ± | 9.77 |  | 57.49 | ± | 10.73 |  | <0.001 |
| Body mass index, kg/m^2^ |  | 25.07 | ± | 4.23 |  | 22.16 | ± | 2.96 |  | 22.11 | ± | 2.80 |  | <0.001 |
| Primary disease, n (%) |  |  |  |  |  |  |  |  |  |  |  |  |  |  |
| Chronic glomerulonephritis |  | 10 (28.6) | | |  | 14 (43.8) | | |  | 12 (33.3) | | |  | 0.700 |
| Diabetes kidney disease |  | 14 (40.0) | | |  | 8 (25.0) | | |  | 11 (30.6) | | |  |  |
| Nephrosclerosis |  | 4 (11.4) | | |  | 4 (12.5) | | |  | 5 (13.9) | | |  |  |
| Polycystic kidney |  | 2 (5.7) | | |  | 0 (0.0) | | |  | 0 (0.0) | | |  |  |
| Other, including unknown |  | 5 (14.3) | | |  | 6 (18.8) | | |  | 8 (22.2) | | |  |  |
| Duration of dialysis, years |  | 6.05 | ± | 5.44 |  | 10.88 | ± | 7.88 |  | 13.57 | ± | 9.83 |  | <0.001 |
| Dialysate calcium concentration, n (%) |  |  |  |  |  |  |  |  |  |  |  |  |  |  |
| 2.5 mEq/L |  | 10 (28.6) | | |  | 9 (28.1) | | |  | 13 (36.1) | | |  | 0.614 |
| 2.75 mEq/L |  | 16 (45.7) | | |  | 13 (40.6) | | |  | 10 (27.8) | | |  |  |
| 3.0 mEq/L |  | 9 (25.7) | | |  | 10 (31.3) | | |  | 13 (36.1) | | |  |  |
| Phosphate binder use, n (%) |  | 33 (94.3) | | |  | 27 (84.4) | | |  | 35 (97.2) | | |  | 0.143 |
| Vitamin D receptor activator use, n (%) |  | 29 (82.9) | | |  | 20 (62.5) | | |  | 27 (75.0) | | |  | 0.169 |
| Prior calcimimetics use, n (%) |  | 14 (40.0) | | |  | 20 (62.5) | | |  | 24 (66.7) | | |  | 0.055 |
| Serum intact parathyroid hormone, pg/mL |  | 350.0  (292.0, 423.0) | | |  | 380.5  (289.0, 484.0) | | |  | 397.5  (332.0, 482.5) | | |  | 0.232 |
| Serum corrected calcium, mg/dL |  | 9.16 | ± | 0.58 |  | 9.43 | ± | 0.73 |  | 9.44 | ± | 0.71 |  | 0.143 |
| Serum phosphate, mg/dL |  | 6.47 | ± | 1.80 |  | 5.64 | ± | 0.97 |  | 5.72 | ± | 0.99 |  | 0.018 |
| Serum corrected calcium x phosphate, mg^2^/dL^2^ |  | 59.352 | ± | 16.892 |  | 53.394 | ± | 11.175 |  | 54.051 | ± | 10.065 |  | 0.122 |
| Serum ALP, U/L |  | 200.0  (167.0, 227.0) | | |  | 252.5  (193.0, 350.0) | | |  | 305.5  (254.5, 397.0) | | |  | <0.001 |
| Serum BAP, µg/L |  | 11.30  (9.30, 12.80) | | |  | 16.65  (14.40, 25.65) | | |  | 20.15  (16.65, 25.70) | | |  | <0.001 |
| Serum total P1NP, ng/mL |  | 225.0  (159.0, 318.0) | | |  | 362.5  (276.0, 421.0) | | |  | 485.5  (360.5, 584.0) | | |  | <0.001 |
| Serum TRACP-5b, mU/dL |  | 384.0  (272.0, 495.0) | | |  | 719.5  (641.5, 765.0) | | |  | 1020.0  (915.5, 1365.0) | | |  | <0.001 |
| Serum BAP/ serum TRACP-5b ratio |  | 0.0281  (0.0253, 0.0364) | | |  | 0.0246  (0.0196, 0.0355) | | |  | 0.0191  (0.0156, 0.0238) | | |  | <0.001 |
| Serum fibroblast growth factor 23, pg/mL |  | 15300  (5140, 34300) | | |  | 6065  (2020, 14100) | | |  | 6930  (936, 12500) | | |  | 0.001 |

Data are shown as number (%), mean ± standard deviation or median (25th percentile, 75th percentile).

Fisher's exact test or one-way ANOVA test was used for comparisons between groups.

ALP, alkaline phosphatase; BAP, bone-specific alkaline phosphatase; P1NP, type 1 procollagen-N-propeptide; TRACP-5b, tartrate-resistant acid phosphatase-5b.

**Supplementary Figure S1**


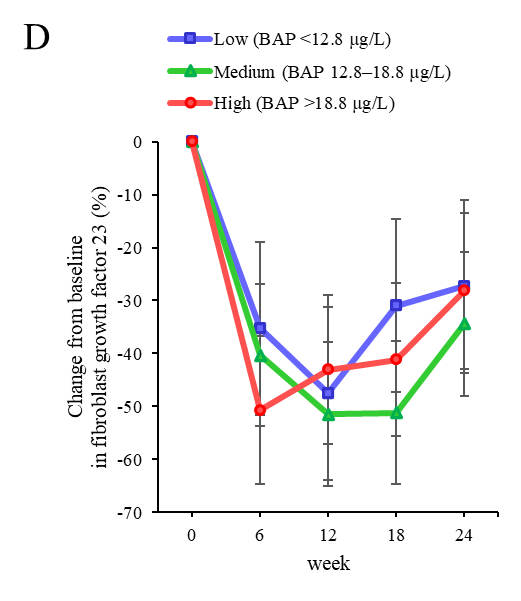

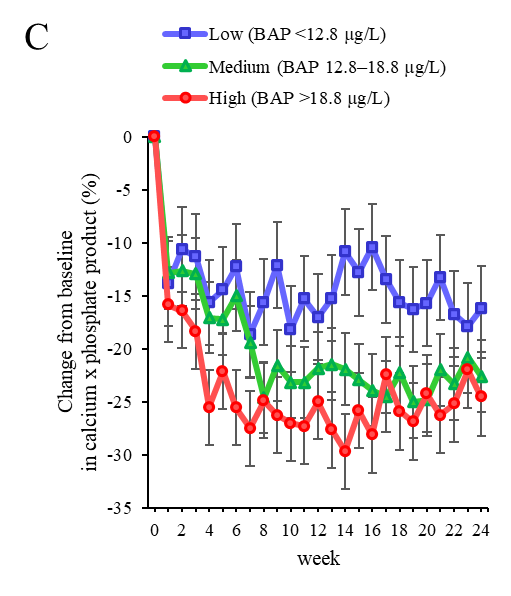

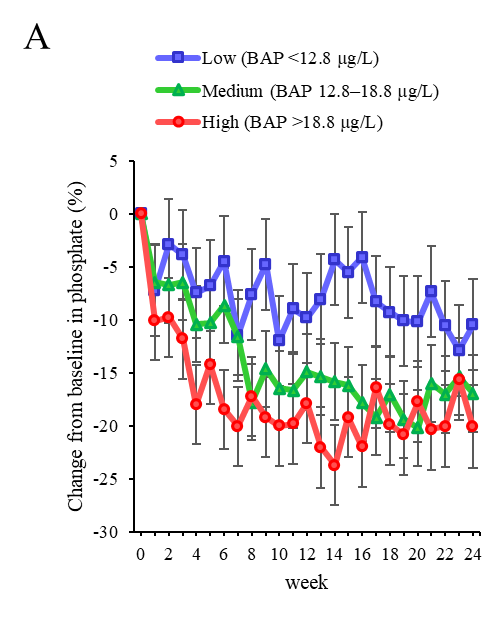
**
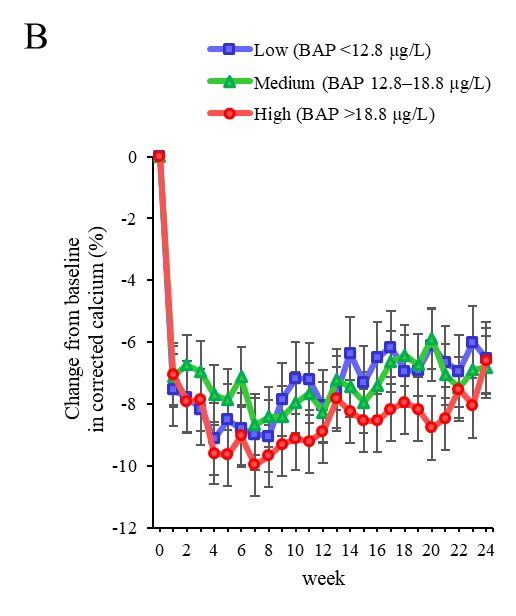
**Percentage changes from baseline in serum levels of P (A), cCa (B), cCa x P product (C), and FGF23 (D) by baseline serum BAP level tertiles in the upacicalcet group.

Data are shown as least square mean ± standard error.

BAP, bone-specific alkaline phosphatase; P, phosphate; cCa, corrected calcium; FGF23, intact fibroblast growth factor 23

**Supplementary Figure S2**


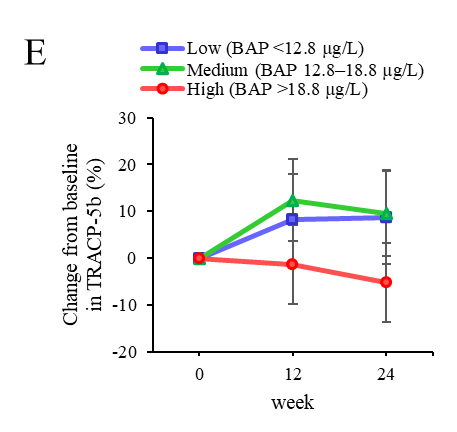

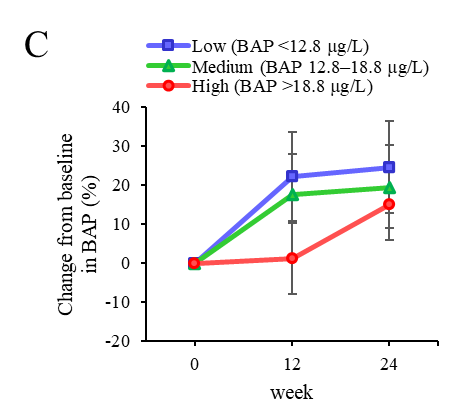

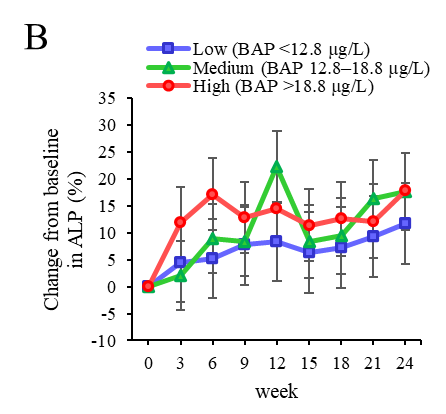

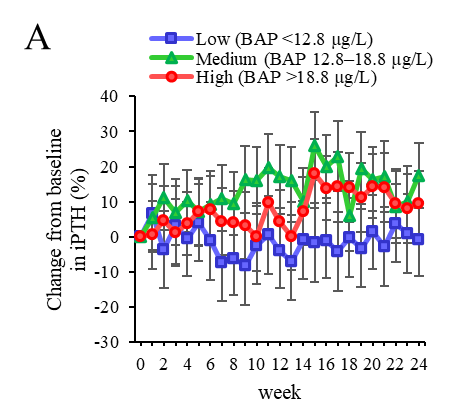

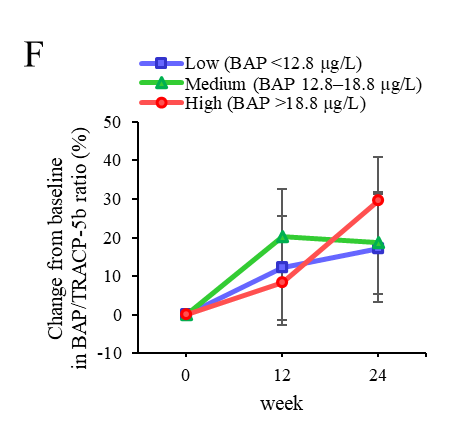

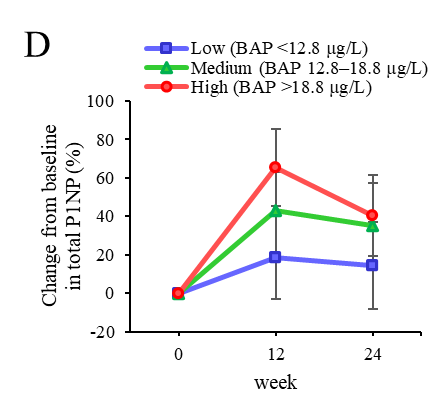
Percentage changes from baseline in serum levels of iPTH (A), ALP (B), BAP (C), total P1NP (D), TRACP-5b (E), BAP/TRACP-5b ratio (F), P (G), cCa (H), cCa x P product (I), and FGF23 (J) by baseline serum BAP level tertiles in the placebo group.


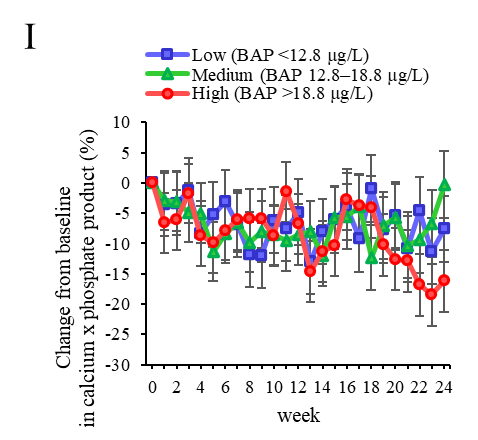

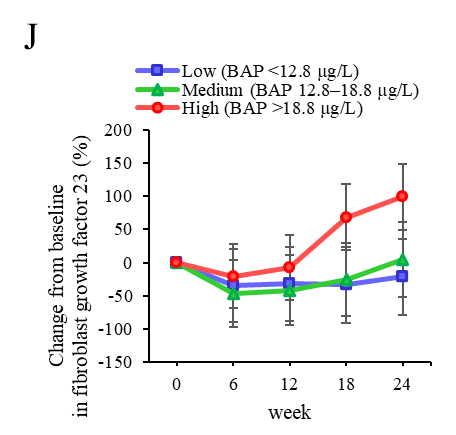
**Supplementary Figure S2 (continued)**


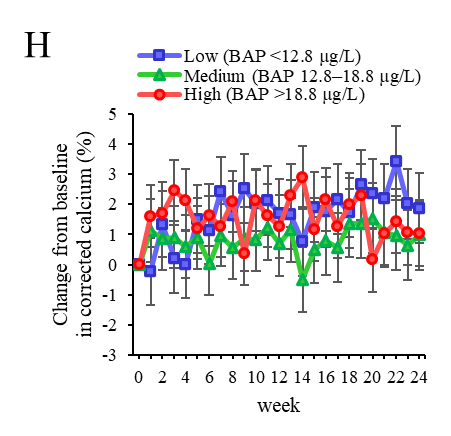

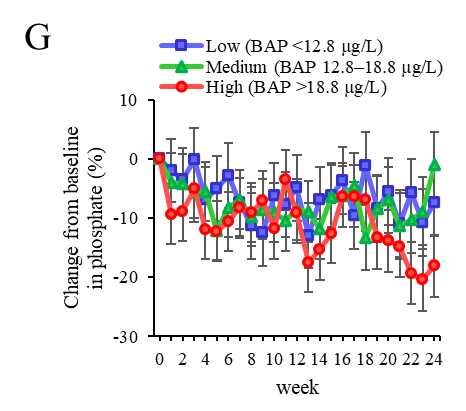


Data are shown as least square mean ± standard error.

BAP, bone-specific alkaline phosphatase; iPTH, intact parathyroid hormone; ALP, alkaline phosphatase; P1NP, type 1 procollagen-N-propeptide; TRACP-5b, tartrate-resistant acid phosphatase-5b; P, phosphate; cCa, corrected calcium; FGF23, intact fibroblast growth factor 23

**Supplementary Figure S3**


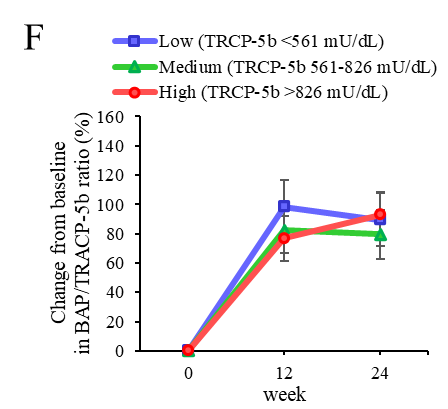

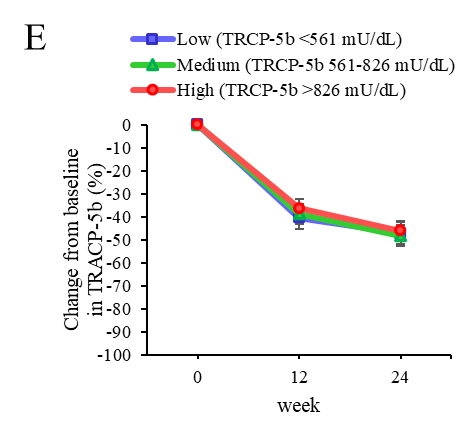

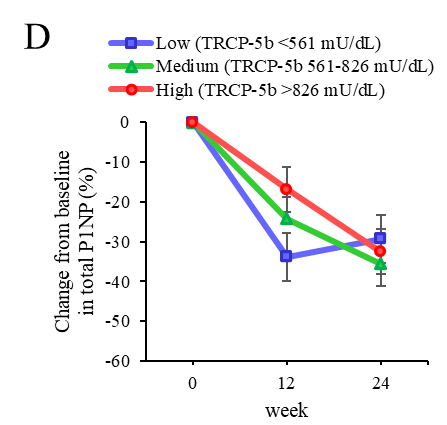

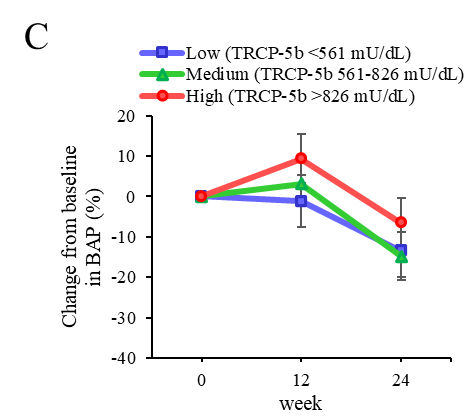
**
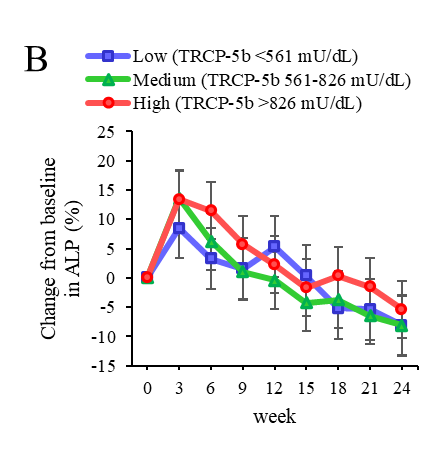
**
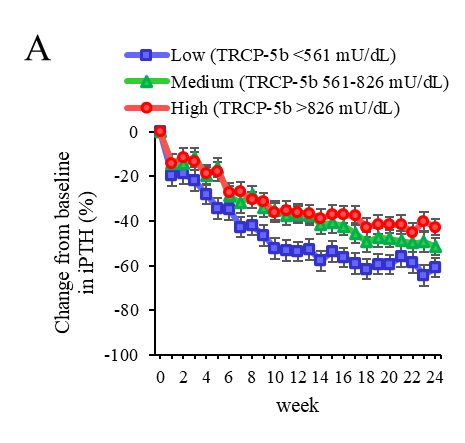
Percentage changes from baseline in serum levels of iPTH (A), ALP (B), BAP (C), total P1NP (D), TRACP-5b (E), and BAP/TRACP-5b ratio (F) by baseline serum TRACP-5b level tertiles in the upacicalcet group.

Data are shown as least square mean ± standard error.

BAP, bone-specific alkaline phosphatase; iPTH, intact parathyroid hormone; ALP, alkaline phosphatase; P1NP, type 1 procollagen-N-propeptide; TRACP-5b, tartrate-resistant acid phosphatase-5b.
